# The synthesis and development of poly(ε-caprolactone) conjugated polyoxyethylene sorbitan oleate-based micelles for curcumin drug release: an *in vitro* study on breast cancer cells†

**DOI:** 10.1039/d3ra03660j

**Published:** 2023-08-04

**Authors:** Nasim Shadmani, Sepehr Gohari, Azin Kadkhodamanesh, Parivash Ghaderinia, Maryam Hassani, Motahare Sharifyrad

**Affiliations:** a Trita Nanomedicine Research & Technology Development Center (TNRTC) Zanjan Health Technology Park Zanjan Iran; b Student Research Center, School of Medicine, Zanjan University of Medical Sciences Zanjan Iran; c School of Pharmacy, Shahid Beheshti University of Medical Sciences Tehran Iran; d Research and Technology Development Center of the Motahar Zist Gostar, Islamic Azad University Zanjan Branch Zanjan Iran 45156-58145 M.rad4294@gmail.com +98 9191815229; e Department of Microbiology, Islamic Azad University Zanjan Branch Zanjan Iran; f Department of Pharmaceutical Biomaterials, Medical Biomaterials Research Center, Faculty of Pharmacy, Tehran University of Medical Sciences Tehran Iran

## Abstract

Background: it is now known that curcumin (Cur) has a broad range of biological properties; however, photosensitivity, as well as low bioavailability and short half-life, have limited its clinical application. To overcome these problems the synthesis of poly(ε-caprolactone)–Tween 80 (PCL–T) copolymers was performed. Methods: the copolymers of PCL–T were created using the solvent evaporation/extraction technique. Then Cur was loaded in PCL–T micelles (PCL–T-M) by a self-assembly method. The characterization of copolymer and micelles was assessed by gel permeation chromatography (GPC), Fourier transform infrared spectroscopy (FT-IR), proton nuclear magnetic resonance spectroscopy (^1^HNMR), differential scanning calorimetry (DSC), transmission electron microscopy (TEM), and dynamic light scattering (DLS) methods. The MTT [3-(4,5-dimethylthiazol-2-yl)-2,5-diphenyltetrazolium bromide] assay was used to indicate the cytotoxicity of the free Cur, PCL–T-M, and Cur-loaded PCL–T-M. Results: TEM analysis showed monodispersed and spherical shapes with a size of about 90 nm. Cur was released from PCL–T-M at pH 7.4 (45%) and 5.5 (90%) during 6 days. After 24 and 48 h, the IC50 of the free Cur, PCL–T-M, and Cur-loaded PCL–T-M on MCF-7 cells were 80.86 and 54.45 μg mL^−1^, 278.30 and 236.19 μg mL^−1^, 45.47 and 19.05 μg mL^−1^, respectively. Conclusion: this study showed that, in the same concentration, the effectiveness of the Cur-loaded PCL–T-M is more than the free Cur, and the nano-system has been able to overcome delivery obstacles of Cur drug. Thus, PCL–T-M can be a candidate as a drug carrier for the delivery of Cur and future therapeutic investigations on breast cancer.

## Abstract 2

The efficacy of PCL–T-M as a drug carrier for the delivery of curcumin against MCF-7 cells and future therapeutic investigations on breast cancer.

## Graphical abstract

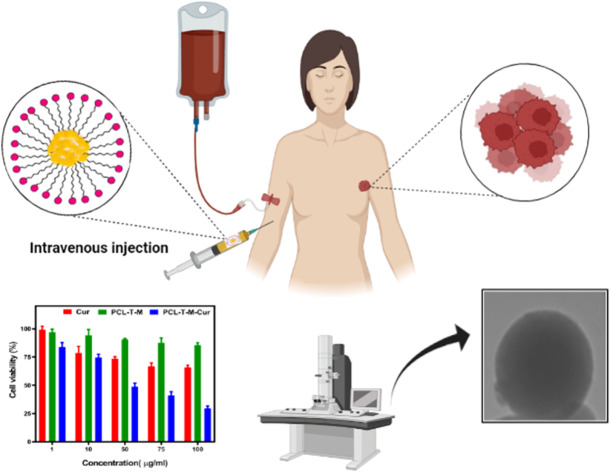

## 1. Introduction

Based on epidemiological analyses, cancer has been reported as one of the major causes of death in the world and the second most common cause of death in the US after heart disease.^1^ The ACS has reported that breast cancer is the most diagnosed malignancy that has the second leading cancer-related mortality in females.^2,3^ For decades, numerous studies have used micelles, lipid-based nanocarriers, silica nanoparticles, and polymeric nanoparticles as drug carriers for the treatment of different types of malignancies.^4^ Nanocarriers are useful in chemotherapy for enhancing the drug accumulation into tumor tissue, improving therapeutic efficacy, decreasing dose, and reducing the systemic toxicity of most drugs. Also, they can increase permeability of the cell membranes, drug solubility, bioavailability, and retention effect in tumor tissue.^5^ The drug delivery systems that have been developed as a strategy for improving insoluble drugs are one of the main focuses of pharmaceutical research and nanotechnology. Besides improved solubility, drug delivery systems can protect the drug and increase its stability, while decreasing its associated adverse effects.^6^ Many studies in the field of polymer-based drug delivery systems have established that in most cases drug's efficacy is enhanced through combining a drug to such a nanosized construction.^7^ Drugs must be released slowly from polymeric micelles for drug targeting. Such accelerated release of drugs from polymeric micelles can potentially lead to precipitation and aggregation of hydrophobic drug inside blood vessels. Moreover, insufficient time may hinder polymeric micelles accumulating at target sites, while slow drug release from polymeric micelles improves target site accumulation and local concentration with minimal drug leakage and premature drug loss. The release rate of drug can be controlled. Copolymers were developed to provide pH-responsive and stimuli-response, including temperature, light, ultrasound, magnetic field, and electronic field, for controlled release of drugs.^8^ Drug-delivery has two targeting approaches. In passive targeting, nanocarriers carrying the drug circulate a sufficient long time in the bloodstream. This prolonged circulation allows accumulation in a desired tissue. On the other hand, in active targeting, specific ligands are attached to the nanocarrier surface to be recognized by receptors expressed by the target cell surfaces. Actively targeted nanoparticulate drug delivery systems can improve the specificity of targeting, reduce systemic toxicity, and increase intracellular release.^9^

Amphiphilic copolymer's self-assembling displays a key role in modern pharmaceutical strategies for anti-neoplastic drugs. Also, it can reduce the drug toxicity in healthy tissues and boost treatment effects by changing the drug pharmacokinetics. Amphiphilic copolymers including mPEG–PCL,^10,11^ mPEG-*b*-PLGA^12^ are used in *in vitro* and *in vivo* biocompatibility studies. Therefore, the multiple properties of polymers can be suitable candidates for biomedical applications, tissue engineering, and medical devices.^13^ Shell crosslinking is an effective strategy to stabilize the micelles but may affect the hydrophilicity of the shell region and surface chemistry.^14^ This strategy potentially enhances the stability of micelles in blood circulation based on the characterization of hydrophilic shells.^15^ Micelles have several properties such as high drug loading capacity and high stability, they are small-sized particles that can inhibit P-glycoprotein, control drug release, hypersensitize towards multidrug-resistant cells, and can be subcellularly localized. Thus, micelles are widely used in pharmaceutical formulations. These formulations can be achieved by attaching specific targeting ligand molecules to the micelle surface.^16^ One of the alternative formulations for the anti-neoplastic drugs is the mPEG–PCL carrier. Although PEG is a bioinert material, it can absorb proteins on its surface and activate complement mechanism. Moreover, its susceptibility to oxidation results in ROS production. Both mechanisms lead to the macrophage chemotaxis,^17^ therefore, T is used to prevent these unwanted reactions.

PCL is a biodegradable hydrophobic and nontoxic applied amphiphilic copolymer.^18^ Therefore, the surface of the copolymers is modified by different materials such as surfactants, polysaccharides, and peptides. T80 is an effective protein stabilizer and solubilizer that is able to transport drugs across biological barriers.^19^ The recent studies reported that while mPEG cannot penetrate deeper parts, T80 and phospholipid–T80 are being used to increase penetration of drugs to BBB. Cao *et al.* investigated encapsulating chlorin e6 and doxorubicin-loaded amphiphilic copolymer PEG-*b*-PCL nanoparticles. Further, they were modified by thioketal for targeting the MDA-MB231 tumors.^20^ Pan *et al.* reported the synthesis of IR780 and paclitaxel-loaded PCL NPs for ovarian cancer treatment by using the phototherapy and chemotherapy studies.^21^

The toxicity effect, immunological influence, and efficiency of macrophage endocytosis in PCL nanoparticles were investigated in recent report.^22^ Ma *et al.* investigated the preparation of docetaxel-loaded PCL–T80 Nps for cellular uptake and cell viability assay into a glioma cell line. PCL–T80 showed higher cellular uptake and cytotoxicity than PCL NPs in C6 glioma cells. The docetaxel-loaded PCL–T80 was then compared with a commercial formulation in the same drug concentration.^23^ Sarpietro *et al.* reported copolymers from PHEA–ethylenediamine (PHEA–EDA) that were modified with polysorbate 80 and polylactide to acquire polymeric micelles of PHEA–EDA–PS_80_–PLA potentially benefit to (*R*)-flurbiprofen release for Alzheimer's disease.^24^ It was concluded that T80 as targeting moiety in the brain causes a prolonged activity of the drug in the cells.^25,26^ The drug delivery systems, consisting of poly ε-caprolactone–Tween 80 copolymers (PCL–T), have opened up a new horizon for the enhancement of drug targeting along with the reduction of adverse effects caused by chemotherapeutic agents.^27^ These types of compounds can be employed for the production of solid spherical nanoparticles, vesicular polymersomes, micelles, polymer–drug conjugates, polyplexes, and dendrimers.^28^ Cur is a polyphenolic compound extracted from the rhizomes of *Curcuma longa*^29^ and is widely used in complementary medicine, as it possesses much biological activity, including analgesic, antioxidant, anti-inflammatory, and antiseptic properties. A large body of evidence shows that Cur has anti-neoplastic activity, and it plays a significant role in regulating the oncogene expression, cell cycle suppression, stimulating the apoptosis process, and preventing tumorigenesis and metastasis.^30–32^ Besides, it has been shown to incite oxidative stress within cancer cells. However, the clinical applications of Cur remain limited because of its short biological half-life, poor solubility resulting in poor absorption, and low bioavailability.^33^ Therefore, novel PCL–T-M micelle was developed as a potential carrier for Cur drugs.

Our study aimed to design, synthesize, and optimize the formulation of PCL–T micelles. Physicochemical properties such as particle size, PDI, morphology, zeta potential, drug loading, and entrapment efficiency were characterized. Moreover, the release behaviors of Cur and PCL–T-M–Cur were determined *in vitro*. The cytotoxic impact of Cur-loaded PCL–T-M was also analyzed at concentration ranges of 1, 10, 50, 75, and 100 μg mL^−1^ against MCF-7 cells using the MTT assay.

## 2. Materials and methods

### 2.1. Chemicals and instrument

The monomeric form of ε-caprolactone, stannous octoate [Sn(OOCC_7_H_15_)_2_], Tween-80, MTT and trypan blue stain were procured from the (Sigma-Aldrich, USA), penicillin/streptomycin, Roswell Park Memorial Institute (RPMI) 1640 cell culture medium, and FBS were obtained from (ATOCEL, Hungary). Cur and all other solvents were purchased from (Sigma-Aldrich, USA).

^1^H NMR (Avance 400, Bruker, Germany), FTIR spectrometer (Tensor 27, Bruker, Germany), DSC (Mettler-Toledo 821, Schwerzenbach, Switzerland), DLS (ZEN3600, Malvern Instruments, Nano ZS, Worcestershire, UK), TEM (EM 208S, Philips, Nederlanden), UV-Vis spectrophotometer (Genesis 10, TERMO, USA), Spectramax microplate reader (Molecular Devices, Sunnyvale, CA), and Gel Permeation Chromatography (GPC) device (GPC 2000, Waters, USA).

### 2.2. Synthesis of PCL–T copolymer

**Fig. 1:**
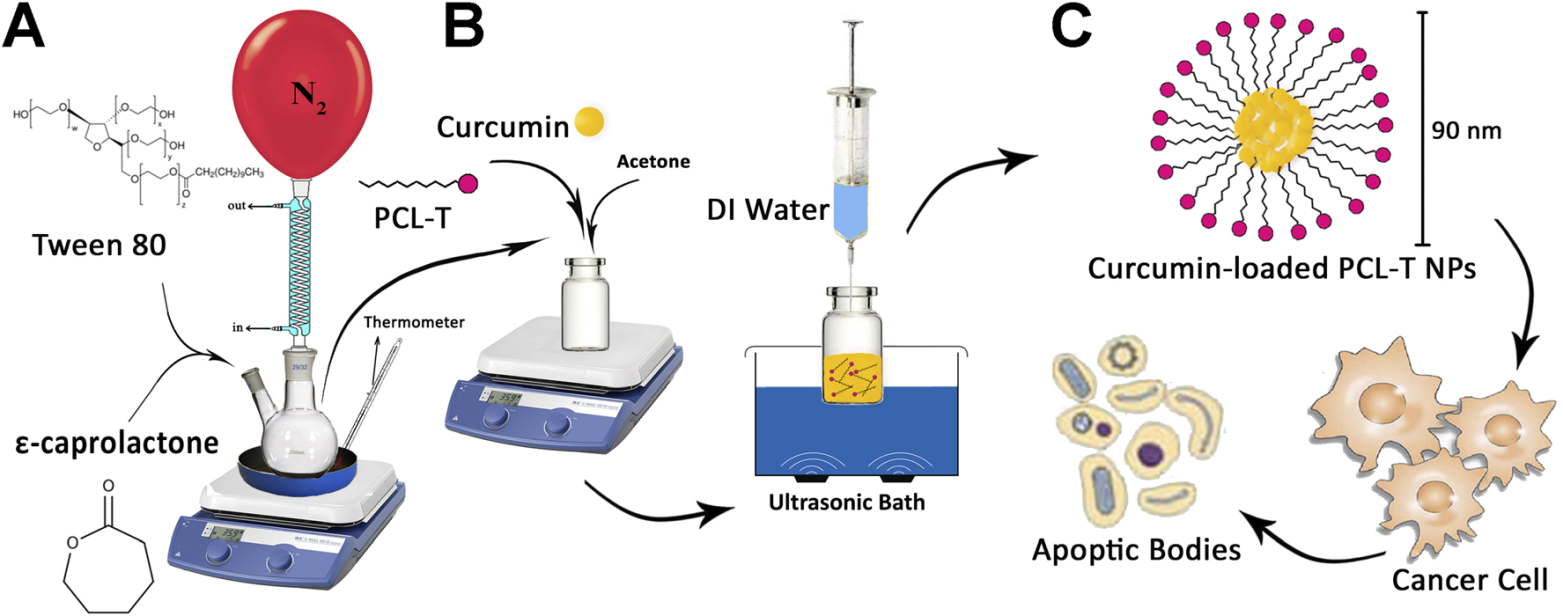
(A) The process synthesis of PCL–T copolymer, (B) drug-loaded PCL–T-M (C), and the cytotoxicity of PCL–T-M–Cur at MCF-7 cell line.

The copolymers of PCL–T were successfully synthesized using T and PCL in the presence of stannous octoate, as a catalyst, which facilitates the ring-opening polymerization process (Fig. 1). In brief, 0.01 mL stannous octoate, 0.5 g Tween 80, and 4.5 g ε-caprolactone were mixed in a flask, and the mixture was heated at 160 °C and left overnight. The synthesis process was conducted in moisture-/oxygen-free conditions. Dichloromethane was used as a solvent to dissolve the resulted product. Next, the obtained mixture was precipitated in extra-cold methanol to eradicate unreacted monomers of Tween 80 and ε-caprolactone (cap). The final product was gathered by the filtration procedures and then lyophilized at 45 °C for 48 h.^33^

### 2.3. Preparation of Cur-loaded PCL–T-M

PCL–T copolymers were synthesized through the solvent evaporation/extraction technique in which acetone was used as a solvent. In brief, copolymers (4 mg) and Cur (1 mg mL^−1^) were dissolved in 500 μL acetone and quickly injected into 5 mL of Mili-Q water (in ultrasonic condition with syringe pressure). The resulting mixture was shaken at a rate of 1000 rpm and magnetically stirred at 25 °C until the organic solvent was completely evaporated. Then EE and DL were measured.^34^ The syntheses of PCL–T copolymer and PCL–T-M and Cur-loaded PCL–T-M are shown in Fig. 1A and B.

### 2.4. Characterization of the PCL–T copolymer, PCL–T-M, and Cur-loaded PCL–T-M

#### 2.4.1. ^1^H NMR analysis

PCL–T copolymer structure was analyzed by ^1^H NMR spectra in CDCl_3_ solution at 400 MHz using the TSM proton signal as a reference.

#### 2.4.2. FTIR analysis

FTIR spectra of samples were performed by a FTIR spectrometer in the range between 4000 and 400 cm^−1^. Typically, dried samples were compressed into KBr pellets for FTIR measurements.

#### 2.4.3. GPC analysis

The average molecular weight (*M*_w_) and the polydispersity (PDI) of polymers were measured by dissolving the sample in tetrahydrofuran (THF) solvent at a flow rate of 1 mL min^−1^ at room temperature using GPC device. The average molecular weight was calibrated by standard polyethylene-glycol with different molecular weights as references. Fifty μL of the sample was injected and analyzed by ‘GPC 2000’ software (Waters, USA).

#### 2.4.4. DSC

In this experiment, approximately 1 mg of samples was employed for DSC analysis using an automatic thermal analyzer. The samples were poured into the sealed aluminum-lead pans and ran at a scan rate of 20 °C min^−1^ initiating from 25 °C and terminating at 150 °C.

#### 2.4.5. Particle size and surface charge

The DLS method was applied to determine the particle size (nm) and zeta potential (mV) of PCL–T-M and PCL–T-M–Cur using a zeta-sizer apparatus.

#### 2.4.6. Morphology

The morphology of PCL–T-M and Cur-loaded PCL–T-M was evaluated by the TEM technique. In this method, a drop of diluted specimens was poured on carbon-coated copper grids to make a thin layer of the liquid film. Afterwards, the excess amounts of solutions were discarded, and the sample was examined with a TEM operating at a voltage of 80 kV.

### 2.5. Physical stability

The stability analysis was measured by assessing the particle size (nm) and zeta potential (mV) of the PCL–T-M and PCL–T-M–Cur in an aqueous dispersion. Upon preparing the micelle dispersion, they were kept at room temperature for 180 days. Then, the size and ZP of PCL–T-M and PCL–T-M–Cur were monitored at time intervals.^14^

### 2.6. Method validating

The maximal absorption of free Cur was detected at a wavelength of 425 nm. Cur showed a linear absorbance at a concentration range between 1 to 7 μg mL^−1^. The LOQ (*N* = 5) and LOD (*N* = 5) of the method were measured at 0.952 and 0.2 μg mL^−1^, respectively.

### 2.7. Drug assay

The Cur content was detected by UV-Vis spectrophotometer (Madison, model GENESYS™ 10S, Thermo Fisher Scientific, USA) at a wavelength of 425 nm in methanol. Standard curves were prepared in the concentration range from 1 μg mL^−1^ to 7 μg mL^−1^ in methanol. As methanol has less volatility than ethanol, it has a standard curve with higher accuracy and precision. For the determination of linear dynamic range, linear regression calibration curves based on eight data points were prepared. The method validation included accuracy, precision, linearity, LOQ, and the LOD. The experimental procedures were repeated 5 times and the obtained values were presented as means ± SD.^34^ The results are shown in ESI Table 1.†

### 2.8. EE and DL

First, Cur-loaded PCL–T-M were centrifuged at 20 000 rpm. Then, both the supernatant and sediment are collected. Based on the initial amount and residual at supernatant of Cur in micelles were calculated using a calibration curve. Briefly, the obtained powder from the freeze dryer of Cur-loaded micelles was weighed and dissolved in methanol. The concentration of Cur at PCL–T-M–Cur in solution was determined by UV-Vis spectrophotometer at a wavelength of 425 nm. DL and EE of the Cur micelles were calculated using the following eqn (1) and (2):1
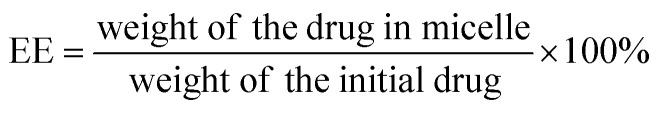
2
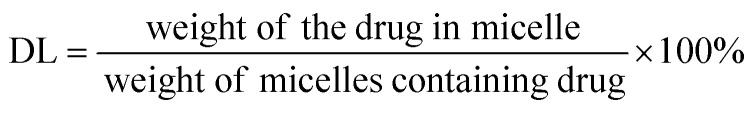


### 2.9. Release profile

The dynamic dialysis method was used to analyze the *in vitro* release profile of Cur from the PCL–T micelles in PBS containing 1% Tween-80 (v/v), as an *in vitro* medium, according to the protocols described previously.^24,25^ First, Cur was dissolved in methanol as the control sample, and the same amounts of Cur micelle suspension or the control solution (equivalent to 1 mg Cur) as well as 1 mL of the release media were mixed and poured into a 12 kDa cut-off dialysis bag. The dialysis bag was immersed in a container containing 100 mL of the same volume of fresh media to evaluate the release rate of Cur from the synthesized micelles using UV-Vis spectrophotometry at a wavelength of 425 nm. The experimental procedures were performed in triplicate. The percentage of accumulative release was calculated and presented as the means ± SD.

### 2.10. Hemolysis test

The blood samples were drawn from healthy individuals and collected in tubes coated with heparin. The specimens were centrifuged at 4000 rpm for 5 min to isolate the plasma samples and separate erythrocytes. The isolated erythrocytes were rinsed with PBS in triplicate. The erythrocyte solution was prepared at a ratio of 1 : 20 with purified erythrocytes and PBS. Then, 500 μL of the prepared solution was mixed into tubes containing PCL–T micelles (5 and 9 mg mL^−1^). As a positive control sample, deionized water was employed (100% hemolysis), while PBS was utilized as a negative control sample (0% hemolysis). All the tubes were gently agitated on a rotary shaker at 37 °C (to resemble the body temperature) for four hours. Tubes were then centrifuged, and the hemoglobin contents were measured by UV-Vis spectrophotometer at a wavelength of 540 nm. The experiments were conducted in triplicate.^35^3
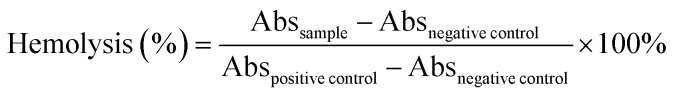


### 2.11. Cytotoxicity of nano-system

The MCF-7 cell line was cultured in the RPMI 1640 cell culture medium containing 10% FBS and 1% penicillin/streptomycin and incubated at 37 °C in a humidified atmosphere containing 5% CO_2_. Next, the cells were harvested by the 0.25% trypsin–EDTA solution from the cell-culture flask and seeded into a 96-well microplate at a density of 10 × 10^5^ cells per well. The MCF-7 cell line was incubated for 24 hours. When the cells reached 70–80% confluence, the cells were treated with different concentrations (1–100 μg mL^−1^) of the free Cur, PCL–T-M, Cur-loaded PCL–T-M for 24, 48 h. For the assessment of the cytotoxicity, 20 μL of the MTT solution (5 mg mL^−1^ in PBS) was added to each well and incubated for 4 h. Subsequently, 100 μL of DMSO was added to each well to dissolve the formazan crystals. The optical density of each well was read at a wavelength of 570 nm using a Spectramax microplate reader. The untreated cells were considered the control cells.

### 2.12. Statistical analysis

Data obtained from the four experiments were reported as mean ± SD. Data sets were analyzed with two-way ANOVA (GraphPad Prism V.5, GraphPad Software, San Diego, CA, USA). For all analyses, statistical significance was done at *α* = 0.05. Significance was measured at *p* < 0.05, *p* < 0.01, *p* < 0.001, and *p* < 0.0001, which were shown with asterisks (*, **, ***, and ****, respectively) or with “ns” to represent no significant difference.

## 3. Results and discussion

### 3.1. Production of nano-system

PCL–T copolymer was synthesized by conjugating T to PCL *via* formation ester group using ring-opening polymerization in the presence of PCL and T (Fig. 1A). In particular, the chosen polymer was PCL due to its biocompatibility, biodegradability, nontoxicity, and insignificant adverse effect. The use of this polymer has been investigated in various biomedical and pharmaceutical fields in previous papers.^36,37^ On the other hand, T was selected as a surfactant for penetration enhancement into cell membrane, though this polymer is potentially useful for targeting cancerous breast tissue. Then, PCL–T-M–Cur is formed according to Fig. 1B.

### 3.2. Characterization of nano-systems

#### 3.2.1. ^1^H-NMR analysis

**Fig. 2:**
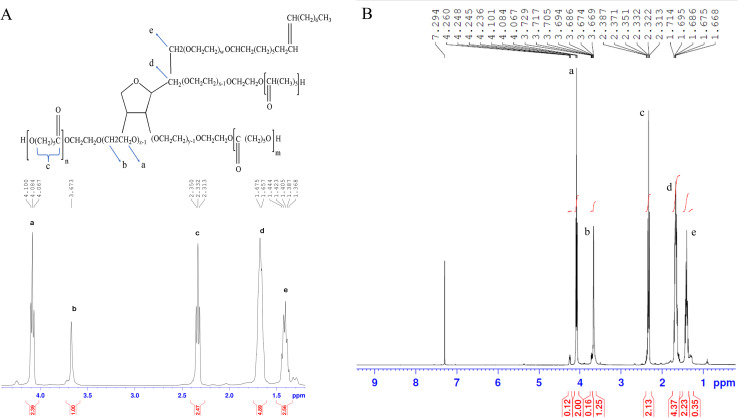
The spectra of ^1^H-NMR belonging to PCL–T copolymer. (A) PCL–T structure and expanded ^1^H-NMR spectrum. (B) The ^1^H-NMR spectrum.

Fig. 2A and B depict the ^1^H NMR spectra of PCL–T dissolved in CDCl_3_. A peak at 3.65 (peak b) corresponds to the protons of the –CH_2_ group belonging to the polyethylene oxide group of T in the copolymer backbone. In the aliphatic region, small peaks belong to the protons of –CH_3_ and –CH_2_ groups in T.^38^ Several peaks at 4.06 (peak a), 2.31 (peak c), 1.60–1.70 (peak d), and 1.35–1.43 (peak e) were assigned to –OCH_2_, –COCH_2_, –CH_2_(3H), and –CH_2_(2H) groups in PCL, respectively.^39^ The analysis demonstrated the synthesis of PCL–T.

#### 3.2.2. GPC analysis

**Fig. 3:**
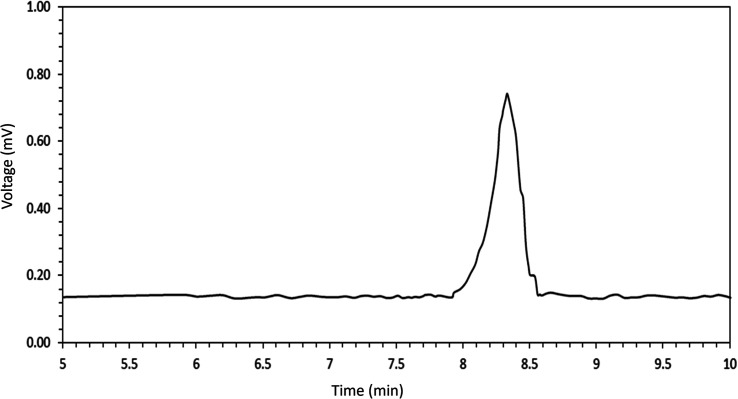
Gel permeation chromatography (GPC) histogram for the assessment of average molecular weight and the polydispersity of the polymers.

According to Fig. 3, the average molecular weight and the PDI of the synthesized co-polymer was measured 17 989/06 Da and 1.234 indicating the successful synthesis of PCL–T co-polymer. The polydispersity indices of the PCL–T copolymer indicate a narrow molecular weight distribution, indicating that no transesterification and/or recoil reactions occurred during the copolymerization.

#### 3.2.3. FT-IR analysis

**Fig. 4:**
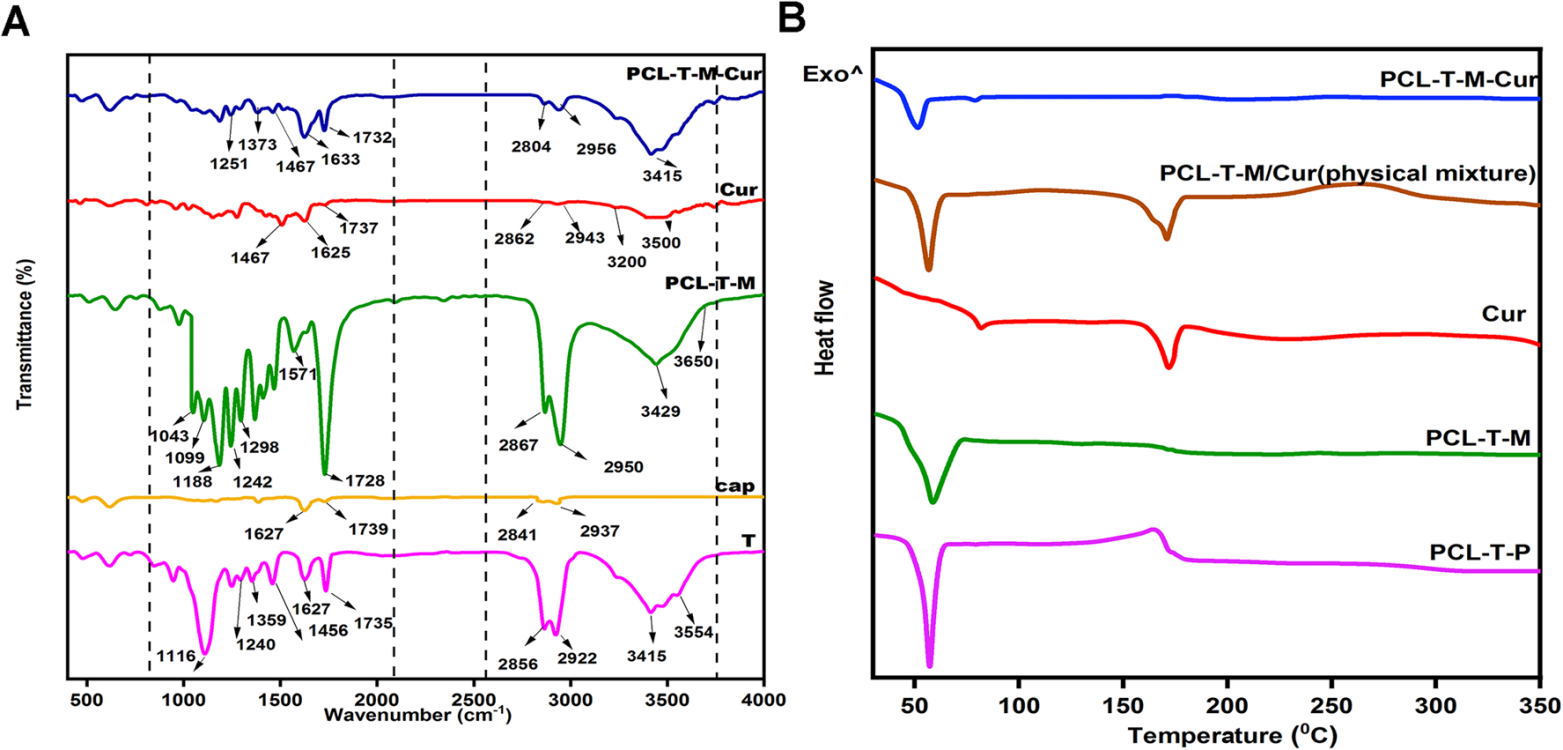
(A) The spectra of FTIR and (B) the DSC curve of the samples.

Fig. 4A shows the FT-IR spectra of T, cap, PCL–T-M, Cur, and PCL–T-M–Cur. T showed a peak at 3400–3500 cm^−1^ due to O–H groups and 1735 cm^−1^ due to carbonyl groups.^40^ The C–H peak at T (CH_2_ symmetric and asymmetric stretches) shows at 2856 and 2922 cm^−1^, respectively. The peaks at 1240 cm^−1^ and 1627 cm^−1^ are associated with the stretching of ester C–O, and bending of CH_2_, respectively. For the cap, the peaks at 1739 cm^−1^ demonstrate carbonyl symmetric at the cap structure.^41^ Peaks at the ranges between 2867 and 2950 cm^−1^ pertain to the stretching band of the –CH_2_ group in the cap structure.^42^ The terminal hydroxyl group of the cap had an absorption band at 3400–3650 cm^−1^, while the peak at 1044–1298 cm^−1^ is assigned to the stretching band group of the C–O.^43^ As shown in PCL–T-M spectra, peaks at 1188 cm^−1^ and 1043–1099 cm^−1^ were attributed to the C–O–C bend of an ester, while at 1242 cm^−1^ corresponds to C–O–C stretching band. The band at 1298 cm^−1^ is used to assess the crystallinity changes of PCL at PCL–T-M.^44^ The analyses indicated the process of PCL–T micelles preparation was accomplished.^23^ FT-IR spectroscopy was performed and the formation of PCL–T-M. PCL–T-M showed a peak at 1728 cm^−1^, which corresponds to the carbonyl group of the micelles. Cur showed peaks at 3200–3500 cm^−1^ due to O–H groups, and a peak at 1467 cm^−1^ for C=C aromatic stretching vibration and the high-intensity band at 1625 cm^−1^ belongs to stretching carbonyl bond vibrations *ν*(C=O).^32^ The appearance of Cur characteristic peaks in the spectrum of PCL–T-M–Cur at 1467, 1633, and 1733 cm^−1^ indicate that Cur has been successfully encapsulated into the micelle. Thus, the intensity peak at 1728 cm^−1^ decreased at PCL–T-M. Therefore, the peak at 1737 cm^−1^ showed combination peak of Cur at PCL–T-M–Cur.

#### 3.2.4. DSC analysis

The DSC analysis (Fig. 4B) demonstrated that the entire synthesized polycaprolactone conjugated Tween 80 co-polymer (PCL–T-P) exhibits a single peak at 60 °C, denoting the created copolymers are devoid of impurities. In Cur-peak at 168 °C, a sharp endothermic peak was observed reflecting the melting point of free Cur and its crystalline nature.^45^ The DSC thermogram of the micelles shows an endothermic transition peak at 60 °C, which can be attributed to the melting point of PCL.^46^ During the loading phase of the drug into the micelle, from 60 °C to 48 °C, the copolymer peak had a left-ward shift in the *T*_m_ indicating the interaction between Cur and PCL–T-M. Moreover, following the encapsulation of Cur into PCL–T-M, the peak at 168 °C disappeared due to the changes from crystalline form to amorphous. These observations signify that Cur has been successfully incorporated into PCL–T-M. The physical mixture of polymer-drug also had an individual peak at the temperature of 168 °C proving the existence of the drug.

#### 3.2.5. Size and potential zeta analysis

**Fig. 5:**
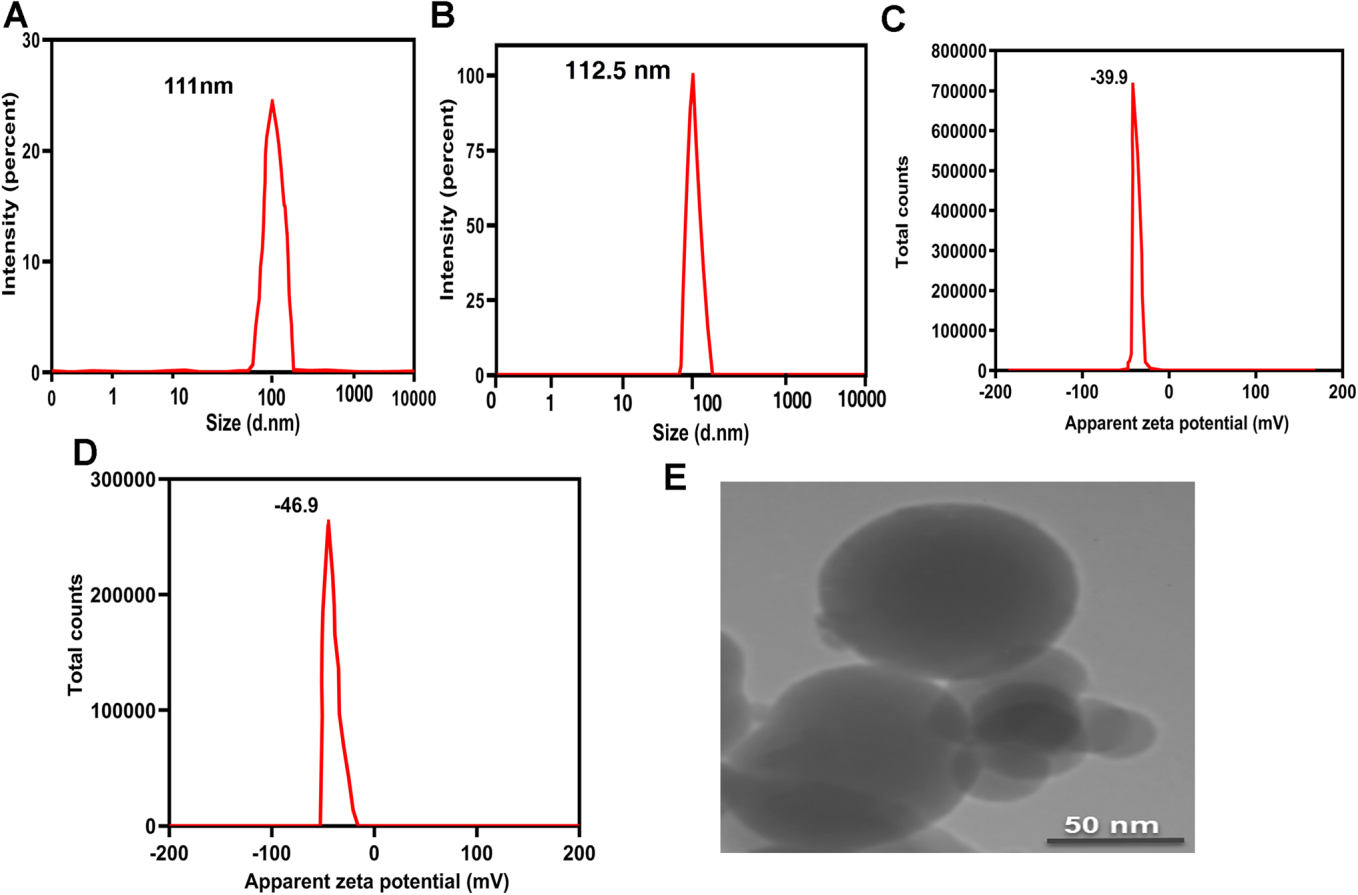
Size of PCL–T-M (A) and PCl–T-M–Cur (B). Zeta potential of PCL–T-M (C) and PCL–T-M–Cur (D). TEM image of PCL–T-M and PCL–T-M–Cur (E). Scale bars are 50 nm.

DLS was used to investigate the size and potential zeta of the PCL–T-M and drug-loaded PCL–T-M. Fig. 5A and B shows the size and PDI of PCL–T-M and Cur-loaded PCL–T. The particle sizes were 111 nm and 112.5 nm with polydispersity of 0.08 and 0.095 for PCL–T-M and Cur-loaded PCL–T-M, respectively, which was slightly bigger than the results obtained by TEM. These results indicate that the micelles were monodispersed and carried a stronger packed core of drug encapsulated in PCL–T-M. There was no significant difference in the size of PCL–T-M before and after the drugs were loaded which demonstrates that Cur was loaded into the PCL–T-M.^47^ The measures of surface charge of PCL–T-M and Cur-loaded PCL–T-M were shown to be about −39.9 mV and −46.9 mV, respectively (Fig. 5C and D). PCL–T-M has R–OCO–R and OH groups and free-electron onto oxygen, which can transfer negative charge on the nano-system surface. The change in the surface charge is the result of Cur loading into PCL–T-M. As Cur possesses electron-rich groups of OH and OCH_3,_ the surface electrostatic potential of PCL–T-M became more negative. Also, based on the results of the study by Paruchuri *et al.* surfactant-Tween 80 may be absorbed on the surface of the polymer's surface with van der Waals bonds in addition to the covalent bonds with caprolactone. As Tween 80 is rich with OH groups, micelles' charge became more negative.^48^

#### 3.2.6. Morphology

TEM micrographs demonstrated particles of monodispersed, uniform size, and spherical shapes of PCL–T-M and PCL–T-M–Cur (Fig. 5E). The particle size of the PCL–T-M was obtained at about 90 nm. Thus, it was slightly smaller than the diameter of DLS.^14^

### 3.3. The physical stability of PCL–T-M and Cur-loaded PCL–T-M

**Fig. 6:**
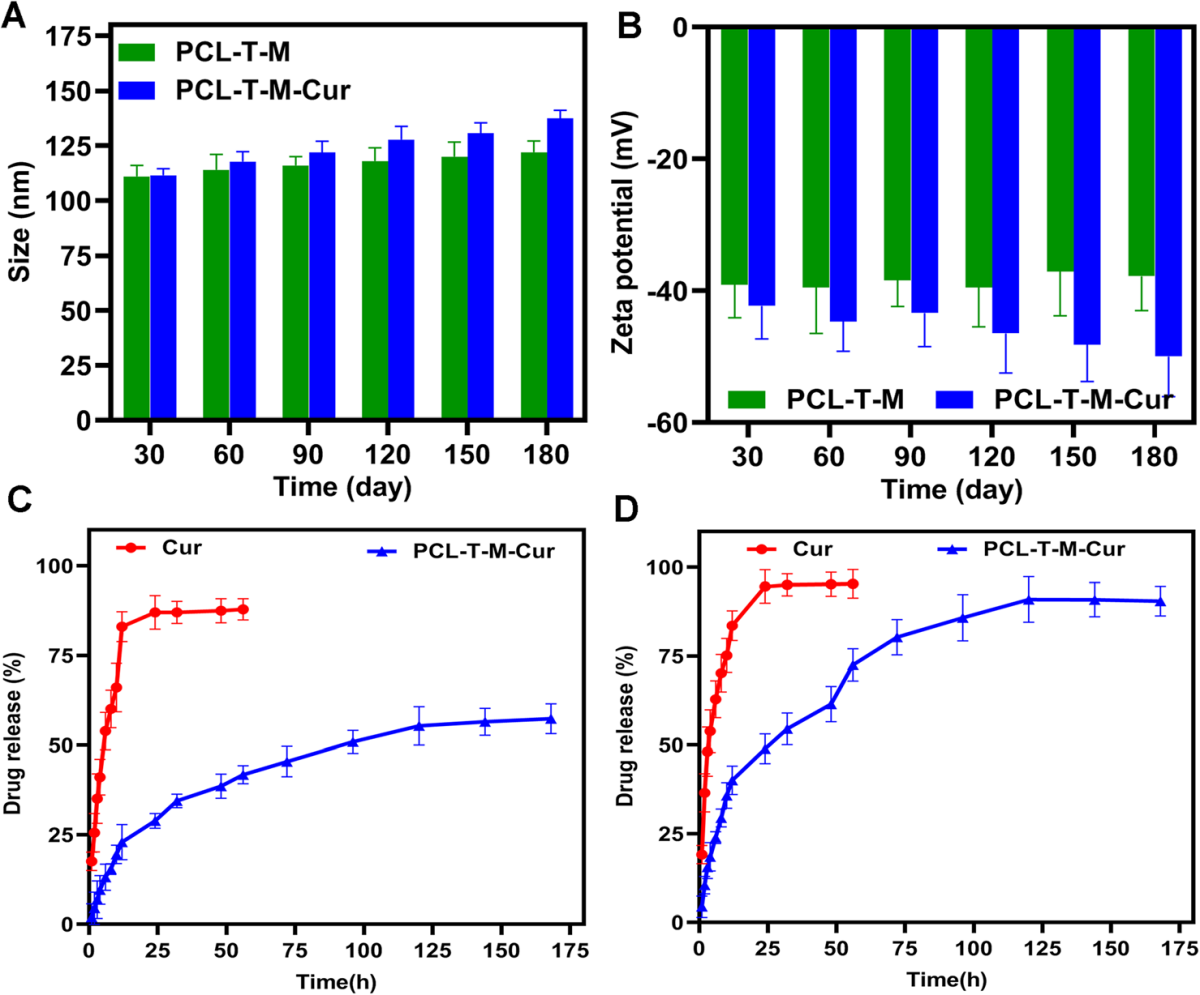
The stability of (A) size (nm) and (B) zeta potential (mV) of PCL–T-M and PCL–T-M–Cur. Release curves of Cur and PCL–T-M–Cur at pH 7.4 (C) and 5.5 (D).

The stability of particle size and zeta potential were monthly monitored for a period of 180 days. Fig. 6A shows some variations in the size of the micelles, demonstrating that the incubation time has a non-significant impact on the particles' dimensions. The analyses indicated that the size and zeta potential of PCL–T-M were stable up to 180 days. The slight increase in the size of micelles during this period can be explained by the hydrophilic nature of T that results in the swelling of the copolymers. As the zeta potential slightly decreased during this time, such an increase in size could not be a sign of aggregation. Moreover, the size and zeta potential of PCL–T-M–Cur was stable up to 90 days; athough, the size and zeta potential were increased through 90 to 180 days (Fig. 6B). The size of the micelle was changed about 10 to 30 nm through 30 to 180 days after loading the drug into the micelle. This phenomenon may be the result of either liberation of the drug from the nano-system or its surfacing on the micelle that are accompanied with increased size and surface zeta potential as well as the micelle aggregation.^49,50^

### 3.4. pH-depended drug release from PCL–T-M

The dialysis method under the sink conditions was applied to evaluate the release profile of Cur from PCL–T-M *in vitro*. In brief, the aqueous solution containing free Cur and the Cur-loaded PCL–T-M (equivalent to 1 mg of Cur) was poured into a dialysis bag (cut-off = 12 kDa). The *in vitro* release analysis was carried out in triplicate for 168 h.^51^Fig. 6C and D show the Cur release behaviors from the PCL–T-M–Cur nano-system over a 168 h period at various PBS solutions with pH 7.4 and 5.5, respectively. For the PCL–T-M–Cur, the release rate of Cur was specifically impressed by pH. The release rates of Cur were slow at pH 7.4. Thus, Cur released about 28% in 24 h and 57% after 168 h from the PCL–T-M–Cur nano-system. Therefore, the drug could be well protected in the PCL–T-M without any obvious burst release. As a result, this PCL–T-M–Cur nano-system had sustained release behavior. In contrast, the drug release was faster at pH 5.5, about 90% after 168 h from the micelle nano-system. In PCL–T-M–Cur, a rapid liberation of Cur was observed at pH 5.5 meaning that the micelle disintegrates at acidic pH which causes stimulus for the release of Cur. This behavior demonstrates the pH-depended of PCL–T-M–Cur. Therefore, Cur shows liberation into cancerous tissue, which can be beneficial in drug delivery against breast cancer. Different kinetic models have been proposed to evaluate release mechanisms of the drug during the releasing period. These models can be used in the assessment of biological efficacy in the drug delivery system.^52^ Different models of drug release from PCL–T-M cargo were studied. The results showed multi-mechanistic drug release of the micelles.^53,54^

The models of drug release analyzed were inclusive first order, Higuchi, zero-order, Weibull, Korsmeyer–Peppas, Logistic, Makoid–Banakar, Hixson–Crowel, and Gompertz for drug release (ESI Table 2†). Each model was approved by regression analysis as slop, intercept, AIC, adjusted *R*-square (*R*_adjusted_^2^), and *R*-square (*R*^2^) in models. AIC is a standard parameter for the evaluation of the kinetics model using maximum probability, that can describe the best fit model using the lowest AIC than the other. Thus, the best fit model was determined by the highest *R*_adjusted_^2^ or *R*^2^ and lowest AIC in a model.^55^ Parameters were performed by the Excel add-in DDSolver program. These results are summarized in ESI Table 3.† The different kinetic equations were used to calculate the release data of Cur from PCL–T-M in both pH 5.5 and 7.4. These results are shown in Tables 1 and 2.† The release data were fitted using kinetic models as Makoid–Banakar, Logistic, Gompertz, and Weibull for PCL–T–Cur micelle at pH 7.4 with *R*^2^ = 0.99. Although, these results were confirmed using Weibull, Logistic and Makoid–Banakar models for PCL–T-M–Cur at pH 5.5 with *R*^2^ = 0.98.

### 3.5. Hemolysis assay

**Fig. 7:**
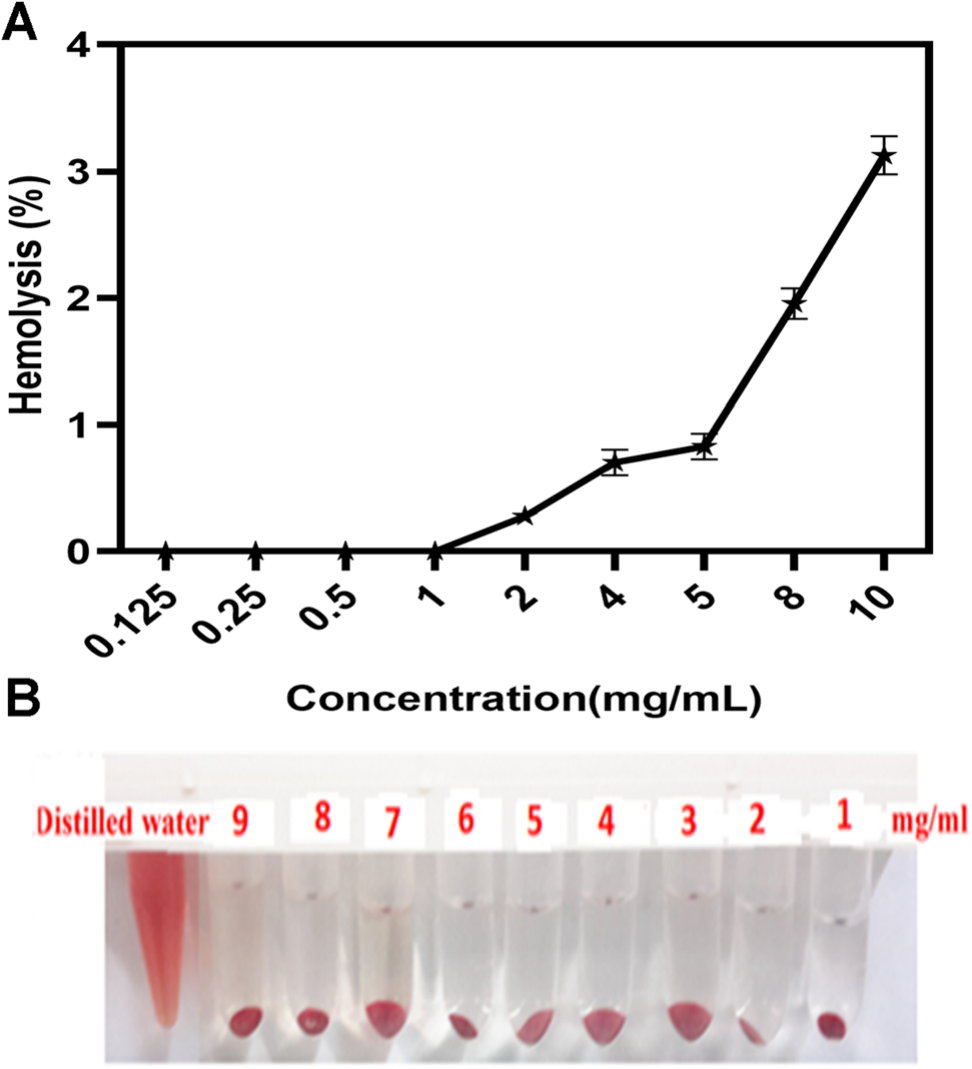
(A) The percentage of hemolysis and (B) schematic of red blood cells show at 1–9 mg mL^−1^ of PCL–T-M after 3 h incubation in 37 °C. Data represent means ± SD deviations (*n* = 6).

Since the synthesized copolymers are supposed to be used for biomedical purposes, it is imperative to determine the biocompatibility of the nanoparticles. Therefore, the hemolysis assay was carried out. Fig. 7A demonstrates that the hemolysis rate was <2%, and synthesized micelles had no hemolytic reactions even at concentrations up to 9 mg mL^−1^. According to international protocols, a hemolytic reaction of less than 3% could be ignored and regarded as a non-hemolytic reaction. This finding is in line with the results of the study of Amin *et al.* in which the safety of Cur-loaded formulations has been assessed, a non-significant hemolysis comprised of 2.65 ± 0.39% and 2.96 ± 3.44% for CP21 and CP51 has been observed, respectively.^56^ Besides, Fig. 7B displays schematic comparisons between copolymers and the control.

### 3.6. Cytotoxicity

**Fig. 8:**
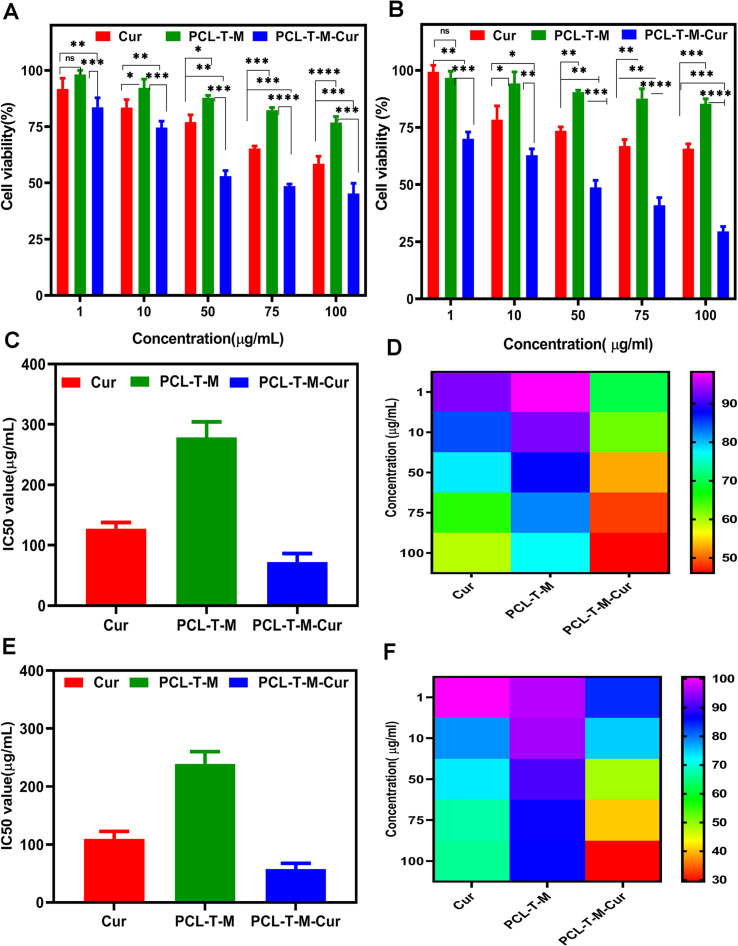
Cell viability of Cur-loaded PCL–T-M on MCF-7 cell lines after (A) 24 and (B) 48 h. IC50 of samples and heatmap of the different concentrations at (C) and (D) 24 h and (E) and (F) 48 h were obtained, respectively.

Fig. 8A and B show cell viability to range of concentration from 1–100 μg mL^−1^ at 24 and 48 h, respectively. According to Fig. 8A, cell viability was determined about 59% and 45% at 100 μg mL^−1^ for Cur and Cur-loaded PCL–T-M at 24 h. The amount of cell viability was measured 65% and 29% at the higher concentration for Cur and Cur-loaded PCL–T-M at 48 h on MCF-7 cell line (Fig. 8B).

The investigated IC50 of the Cur-loaded PCL–T-M on MCF-7 cells after 24 and 48 h were about 45.47 and 19.05 μg mL^−1^, respectively. The IC50 of free Cur was calculated to be 80.86 and 54.45 μg mL^−1^ after 24 and 48 h, which indicates the IC50 of Cur-loaded PCL–T-M was lower than free Cur on MCF-7 cells (Fig. 8C and E). The IC50 of PCL–T-M measurements were 278.30 and 236.19 μg mL^−1^, respectively, and PCL–T-M did not show toxic traces.^57–59^ The heatmap of concentration of Cur-loaded PCL–T-M, free Cur, and free PCL–T-M show using color changes from red (low intensity) to violet (high intensity), respectively (Fig. 8D and F).

The results showed that Cur-loaded PCL–T-M had a more lethal effect on cancer cells than free Cur. In fact, micelles are much easier to absorb than the free drug, and the drug can enter the interior of the cell and exert its effect. In this way, the lipophilic and relative instability problems of Cur drug can be solved using nano-system. The results show that in both cases, increasing the drug dosage is associated with more lethality in cancer cells and that the effectiveness is dose dependent. In addition, when there is more contact within the cells, the lethality increases. Also, we observed a time-dependent regional effect. In 48 h, the lethality was higher than in 24 h in all cases, which was significantly different compared to the control case.

## 4. Conclusion

In this study, we successfully synthesized and self-assembled micelles for the drug delivery of Cur for the treatment of breast cancer. The *in vitro* analyses revealed that the synthesized copolymers were biologically safe and caused no remarkable toxicity. The release profile of PCL–T-M–Cur showed that the release pattern is pH-dependent and has sustained release system for Cur into the tumor region and the tumor environment. The MTT assay showed that PCL–T-M–Cur significantly decreased the cell viability in MCF-7 cell line compared with the free Cur and enhanced antitumor activity. Therefore, our findings demonstrated that the synthesized PCL–T-M–Cur can be a potential therapeutic for targeting breast cancer cells in the future.

## Abbreviations

ACS: American Cancer Society
PCL–T: Poly(ε-caprolactone)–Tween 80
Cur: Curcumin
T: Tween 80
PCL–T-M: PCL–T micelles
Cap: ε-Caprolactone
PCL–T-P: Poly(ε-caprolactone)–Tween 80 polymer
BBB: Blood brain barrier
PDI: Poly-dispersity index
ZP: Zeta potential
DL: Drug-loading
EE: Entrapment efficiency
FBS: Fetal bovine serum
GPC: Gel permeation chromatography
FTIR: Fourier-transform infrared spectroscopy
^1^HNMR: Proton nuclear magnetic resonance spectroscopy
THF: Tetrahydrofuran
DSC: Differential scanning calorimetry
TEM: Transmission electron microscopy
DLS: Dynamic light scattering
mPEG–PCL: Methoxy poly(ethylene glycol)–poly(ε-caprolactone)
mPEG-*b*-PLGA: Polymethoxy ethylene glycol–polylactic-*co*-glycolic acid
MTT: [3-(4,5-Dimethylthiazol-2-yl)-2,5-diphenyltetrazolium bromide]
RPMI 1640: Roswell Park Memorial Institute 1640
LOQ: Limits of quantitation
LOD: Limits of detection
PBS: Phosphate buffered saline
AIC: Akaike information criterion

## Ethical statement

This project has been approved by the ethic committee of Islamic Azad University, Zanjan branch, Iran.

## Data availability

The datasets used and/or analyzed during the current study are available from the corresponding author on reasonable request.

## Author contributions

N. S.: conceptualization, data curation, formal analysis, investigation, methodology, software, validation, visualization, writing (original draft). S. G.: conceptualization, investigation, writing (editing), revision. A. Z.: methodology. P. G.: methodology, validation. M. H.: methodology, software, validation. M. S. R.: conceptualization, data curation, formal analysis, methodology, validation, supervision, visualization, writing (original draft), writing (editing), revision, funding, project administration.

## Conflicts of interest

The authors contributing to the study have nothing relevant to disclose.

## Supplementary Material

RA-013-D3RA03660J-s001
