# Supplementary material for: The synthesis and development of poly(ε-caprolactone) conjugated polyoxyethylene sorbitan oleate-based micelles for curcumin drug release: an in vitro study on breast cancer cells
Source: RSC Adv. 2023 Aug 4;13(34):23449–60. doi: 10.1039/d3ra03660j (PMC10401665; doi:10.1039/d3ra03660j)
Supplement: RA-013-D3RA03660J-s001 [file RA-013-D3RA03660J-s001.pdf]

Electronic Supplementary Material (ESI) for RSC Advances.
This journal is © The Royal Society of Chemistry 2023

Supplementary file

Table 1: accuracy and precision of UV-Vis spectroscopy method for curcumine

Standard solution concentration (µg/mL)	Accuracy	Precision (RSD %)
1	88.63849765	5
2	109.4209703	5.329774327
3	102.0552947	6.942986042
4	101.971831	6.555547774
5	100.9827856	3.851956345
6	98.23682838	6.77115196
7	99.40532081	3.566206767

Table 2. The type of kinetics equation explained the mechanism release of Cur from the PCL-T-M nano-system.

Model	Equation
First-order	$F=100\times[1-Exp(-k_1\times t)]$
zero-order	$F=F_0+k_0\times t$
Higuchi	$F=F_0+kH\times t^{0.5}$
Korsmeyer-Peppas	$F=F_0+kKP\times t^n$
Hixson-Crowel	$F=100\times[1-(1-kHC\times t)^3]$
Makoid-Banakar	$F= kMB\times t^n\times Exp(-kt)$
Weibull	$F=100\times\{1-Exp[-(t\text{^}\beta)/\alpha]\}$
Logistic	$F=100\times Exp[\alpha+\beta\times log(t)]/\{1+Exp[\alpha+\beta\times log(t)]\}$
Gompertz	$F=100\times Exp\{-\alpha\times Exp[-\beta\times log(t)]$

Table 3. The parameter of the kinetics model at pH 7.4 and 5.5 from PCL-T-M-Cur.

Model	Parameter	PBS (7.4)	PBS (5.5)
First-order	k_1	0.008	**0.026**
	R^2	0.784	**0.929**
	AIC	123.91	**120.41**
Zero-order	k_0	0.461	**0.757**
	R^2	0.551	**0.410**
	AIC	138.39	**156.58**
Higuchi	kH	5.049	**8.382**
	R^2	0.964	**0.935**
	AIC	93.43	**119.04**
Korsmeyer-Peppas	kKP	6.704	**13.037**
	n	0.436	**0.4**
	R^2	0.973	**0.9652**
	AIC	88.99	**109.35**
Hixson-Crowell	kHC	0.002	**0.007**
	R^2	0.720	**0.887**
	AIC	128.361	**128.453**
Makoid-banakar	kMB	4.495	**8.849**
	n	0.600	**0.563**
	k	0.003	**0.003**
	R^2	0.992	**0.988**
	AIC	67.659	**91.082**
Weibull	α	18.04	**12.401**
	β	0.553	**0.668**
	R^2	0.987	**0.988**
	AIC	77.111	**91.682**
Logistic	α	-3.092	**-2.926**
	β	1.556	**2.223**
	R^2	0.993	**0.980**
	AIC	65.45	**99.47**
Gompertz	α	4.158	**4.655**
	β	0.904	**1.519**
	R^2	0.996	**0.963**
	AIC	56.66	**110.105**
